# Emerging Two-Dimensional Ti3C2-BiOCl Nanoparticles for Excellent Antimicrobial and Antioxidant Properties

**DOI:** 10.7759/cureus.65080

**Published:** 2024-07-22

**Authors:** V. RA. Rathina Gesav, A. Geetha, S. Vasugi, S. Balachandran, I.G.K. Ilangovar

**Affiliations:** 1 Department of Physiology, Saveetha Dental College and Hospitals, Saveetha Institute of Medical and Technical Science, Saveetha University, Chennai, IND

**Keywords:** bovine serum albumin, x-ray diffraction, titanium aluminium carbide, bismuth oxychloride, mxenes

## Abstract

Introduction

MXenes (Ti_3_C_2_) represent a group of two-dimensional inorganic compounds, produced through a top-down exfoliation method. They comprise ultra-thin layers of transition metal carbides, or carbonitrides, and exhibit hydrophilic properties on their surfaces. Utilizing Ti_3_C_2 _BiOCl nanoparticles for their antimicrobial and antioxidant attributes involves enhancing synthesis, processing, and characterization techniques.

Materials and method

To prepare Ti_3_C_2_ MXene, dissolve 1.6 g of LiF in 20 ml of 9M HCl. Slowly add 1 g of Ti_3_AlC_2_ (titanium aluminum carbide) powder to the solution while stirring. Etch at 35°C for 24 h to remove Al layers from Ti_3_AlC_2_, leaving Ti_3_C_2_ layers. Wash the mixture with distilled water and ethanol until the pH is around 6. Collect the washed sediment by centrifugation and sonicate it in distilled water for 1 h. Centrifuge to remove unexfoliated particles. For BiOCl synthesis, dissolve 2 mmol of Bi(NO_3_)_3_·5H_2_O (bismuth nitrate pentahydrate) in 10 ml of 2M HCl (hydrochloric acid) with 0.5 g of PVP (polyvinylpyrrolidone). Transfer the solution to a Teflon-lined autoclave, fill it with distilled water up to 80%, and heat at 160°C for 24 h. Collect the precipitate by centrifugation, wash, and dry at 60°C for 12 h. Disperse BiOCl nanoparticles in distilled water, sonicate for 30 min, add Ti_3_C_2_ MXene dispersion, stir for 2 h, collect, wash, dry, and calcine at 400°C for 2 h.

Result

The Scanning Electron Microscope (SEM) utilizes electrons, rather than light, to generate highly magnified images. Energy Dispersive X-ray Spectroscopy (EDS) complements SEM by analyzing the X-ray spectrum emitted when a solid sample is bombarded with electrons, enabling localized chemical analysis. In SEM imaging, incorporating an X-ray spectrometer allows for both element mapping and point analysis. The SEM image of the prepared samples reveals accordion-like multilayer structures in BiOCl, characterized by thin sheet-like structures with numerous pores. EDS, relying on X-ray emissions from electron bombardment, facilitates detailed chemical analysis at specific locations within the sample.

Conclusion

Our research has shed light on the synthesis and characterization processes of two-dimensional Ti_3_C_2 _BiOCl nanoparticles, revealing their remarkable antimicrobial and antioxidant properties.

## Introduction

MXenes (Ti_3_C_2_) represent a class of two-dimensional inorganic compounds initially outlined and employing a top-down exfoliation technique for synthesis. These compounds comprise atomically thin layers of transition metal carbides or carbonitrides, exhibiting hydrophilic behavior on their surfaces [1]. The extraction of the "A" group layer from MAX phases leads to the formation of 2-D layers termed Ti_3_C_2_. Since their discovery, numerous researchers have delved into the properties and applications of Ti_3_C_2_ across diverse fields, encompassing energy storage, sensing, catalysis, and electronics [2]. Notably, Ti_3_C_2_ are distinguished by their elevated electrical conductivity and expansive surface area, rendering them auspicious candidates for energy storage applications such as supercapacitors and Li-ion batteries. The capability of Ti_3_C_2_ electrodes to notably enhance supercapacitor performance, exhibiting high specific capacitance and remarkable stability [3]. Beyond energy storage, Ti_3_C_2_ exhibits considerable promise in realms like sensing and catalysis. A single layer of Ti_3_C_2_ serves as a highly sensitive and selective gas sensor for NO_2_ detection. Similarly, Ti_3_C_2_ emerges as a highly active catalyst across various reactions [4,5].

Bismuth oxychloride (BiOCl) is a chemical compound utilized across various industries, including cosmetics, pharmaceuticals, and pigments. This white, crystalline powder exhibits a metallic sheen and serves as a cost-effective alternative to more expensive metals like titanium and iron. BiOCl, discovered by French chemist Louis Nicolas Vauquelin in 1789, has garnered significant attention for its distinctive properties and diverse applications. In cosmetics, it functions as a pearlescent pigment, imparting a shimmering effect by scattering light [6].

BiOCl showcases pharmaceutical potential, with a focus on its antimicrobial and antioxidant attributes. Its application spans various forms, including creams and ointments, addressing skin ailments like acne and fungal infections. Furthermore, BiOCl serves as a coating material for drug delivery systems, safeguarding drugs from degradation owing to its biocompatibility [7]. The introduction of Ti_3_C_2_-BiOCl presents an intriguing avenue for combating a broad spectrum of microorganisms, potentially paving the way for antimicrobial coatings, medical devices, or disinfectants. The amalgamation of Ti_3_C_2_ and BiOCl may yield materials endowed with noteworthy antioxidant properties, offering avenues for mitigating oxidative stress-related diseases in medicine [8]. Notably, Ti_3_C_2_ catalytic prowess could be further enhanced or modified by the inclusion of BiOCl, rendering the composite material adept for catalyzing chemical reactions. Leveraging Ti_3_C_2_'s extensive surface area, applications in energy storage devices, catalysis, and sensors are plausible. BiOCl's photocatalytic prowess, when synergized with Ti_3_C_2_, might amplify environmental remediation efforts, facilitating water purification or air treatment. If the Ti_3_C_2_-BiOCl composite proves biocompatible, its potential in biomedical domains, such as drug delivery or imaging agents, could be transformative. Moreover, the addition of BiOCl to Ti_3_C_2_ enhances the composite's mechanical properties, augmenting its utility in applications mandating robustness and durability [9].

Ti_3_C_2_ typically demonstrates thermal stability, but the addition of BiOCl may alter the composite material's thermal behaviour, rendering it suitable for high-temperature environments. Ti_3_C_2_-BiOCl could manifest distinctive electronic and optical properties, facilitating their application in electronic devices, sensors, or optoelectronic devices. The synthesis and utilization of Ti_3_C_2_-BiOCl for its antimicrobial and antioxidant attributes have garnered attention, with research indicating certain Ti_3_C_2_ variants effectively inhibiting bacterial and fungal growth, thus holding promise for antimicrobial and antioxidant applications [10]. For instance, a study by Chen et al. showcased Ti_3_C_2_, MXene's potent antibacterial activity against both Gram-negative and Gram-positive bacteria. Beyond antimicrobial effects, Ti_3_C_2_ exhibits antioxidant activity, mitigating oxidative stress by scavenging free radicals, owing to their high surface area and conductivity. Similarly, bismuth oxychloride (BiOCl) possesses antimicrobial and antioxidant properties, inhibiting various bacteria and fungi, including antibiotic-resistant strains, while effectively neutralizing free radicals to mitigate oxidative stress. In summary, Ti_3_C_2_ and BiOCl exhibit antimicrobial and antioxidant potentials, positioning them as versatile candidates across diverse fields such as biomedicine, cosmetics, and food science. The study aims to explore the utilization of Ti_3_C_2_-BiOCl nanoparticles for their antimicrobial and antioxidant properties, alongside strategies to enhance synthesis, processing, and characterization methodologies [11].

## Materials and methods

Materials

The study materials included the following: titanium aluminum carbide (Ti_3_AlC_2_), Hydrochloric acid (HCl), Lithium fluoride (LiF), Hydrogen fluoride (HF), Distilled water, Ethanol, Bismuth nitrate pentahydrate (Bi(NO_3_)_3_·5H_2_O), Hydrochloric acid (HCl), Polyvinylpyrrolidone (PVP), Teflon-lined stainless steel autoclave and centrifuge.

Preparation of Ti_3_C_2_ (MXene)

To prepare Ti_3_C_2_ MXene, titanium aluminum carbide (Ti_3_AlC_2_) is used as the precursor. First, 1.6 g of lithium fluoride (LiF) is dissolved in 20 mL of 9M hydrochloric acid (HCl) solution. Then, 1 g of Ti_3_AlC_2_ powder is slowly added to the solution while stirring continuously. This reaction is carried out at 35°C for 24 h to etch out the aluminum layers from Ti_3_AlC_2_, leaving behind the Ti_3_C_2_ layers. After etching, the resulting mixture is washed several times with distilled water and ethanol until the pH of the supernatant reaches around 6, indicating the removal of excess acid and salts. The washed sediment is collected by centrifugation. Next, the sediment is dispersed in distilled water and sonicated for 1 h to delaminate the Ti_3_C_2_ layers into single or few-layer sheets. The dispersion is then centrifuged to remove any unexfoliated particles [12].

Synthesis of BiOCl

For the synthesis of BiOCl, bismuth nitrate pentahydrate (Bi(NO_3_)_3_·5H_2_O), hydrochloric acid (HCl), polyvinylpyrrolidone (PVP), and distilled water are used. First, 2 mmol of Bi(NO_3_)_3_·5H_2_O is dissolved in 10 mL of 2M HCl solution, and 0.5 g of PVP is added to the solution, stirring until completely dissolved. This solution is then transferred into a Teflon-lined stainless steel autoclave, which is filled with distilled water up to 80% of its capacity. The autoclave is sealed and heated at 160°C for 24 h. After cooling to room temperature, the resulting precipitate is collected by centrifugation, washed with distilled water and ethanol, and then dried at 60°C for 12 h [13].

Synthesis of Ti_3_C_2 _BiOCl composite nanoparticles

To form the Ti_3_C_2 _BiOCl composite, a certain amount of BiOCl nanoparticles is dispersed in distilled water and sonicated for 30 min to ensure uniform dispersion. The previously prepared Ti_3_C_2_ MXene dispersion is then added to the BiOCl solution under continuous stirring. The mixture is stirred for 2 h to ensure that the Ti_3_C_2_ uniformly coats the BiOCl nanoparticles. The composite is collected by centrifugation, washed with distilled water, and dried at 60°C for 12 h. To improve the crystallinity and stability of the composite, the dried product is calcined at 400°C for 2 h under an inert atmosphere, such as nitrogen or argon [14].

Statistical analysis

The means of standard deviation were used to display the findings of three separate experiments. Every study was conducted twice. The statistical analysis used a one-way analysis of variance, and a result was considered statistically significant if its p value was 0.05 or above.

## Results

X-ray diffraction (XRD) analysis

**Figure 1:**
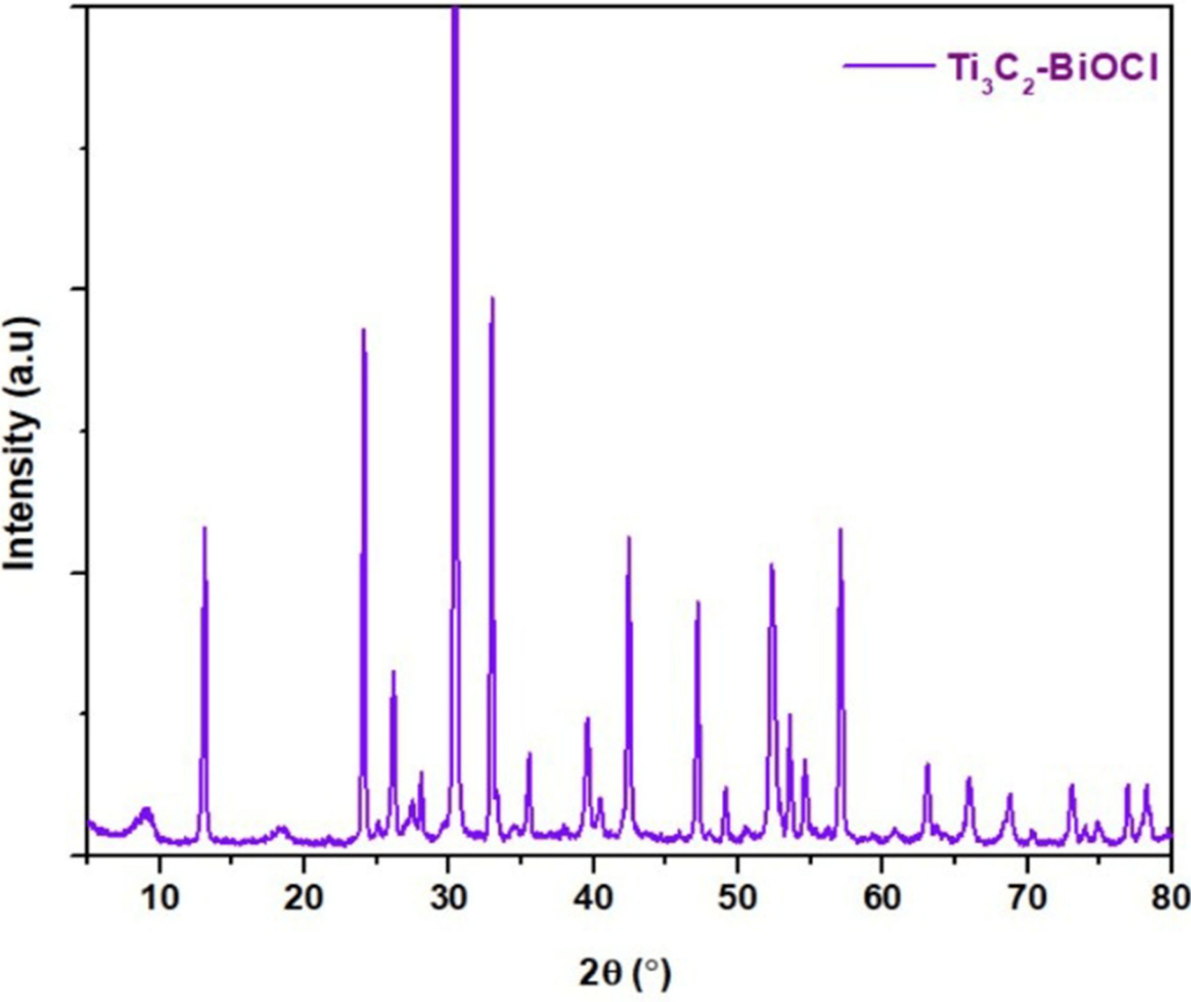
XRD Analysis of Ti3C2-BiOCl XRD: X-ray Diffraction, Ti_3_C_2_-BiOCl: Titanium Carbide(MXene)-Bismuth Oxychloride

The XRD analysis of our synthesized material revealed characteristics of both Ti_3_C_2_ and BiOCl, demonstrating a high degree of crystallinity. Upon comparing the XRD pattern of Ti_3_C_2_-BiOCl with that of Ti_3_AlC_2_, peaks were observed at 9°, 19°, 39°, 42°, 56°, and 69°. Furthermore, when compared with the standard pattern from JCPDS card 06-0249, additional peaks were noted at 13°, 24°, 26°, 31°, 36°, 47°, and 49°. All the diffraction peaks were sharp and intense, and no impurity peaks were measured which indicated a good crystallinity of the as-synthesized sample. It was to be noted that the diffraction peak ratio values of (101), (102), and (110) were higher than (001) which implied that the BiOCl slabs preferred to grow along the (101), (102) and (110) orientations, leading to highly exposed (001) facets. Calculated inter-layer distances for Ti_3_AlC_2_ and Ti_3_C_2_-BiOCl were found to be 0.92 nm and 1.02 nm, respectively. This increase in the interlayer distance for Ti_3_C_2_ confirms the successful etching effect (Figure 1).

Scanning electron microscopy (SEM) analysis

**Figure 2:**
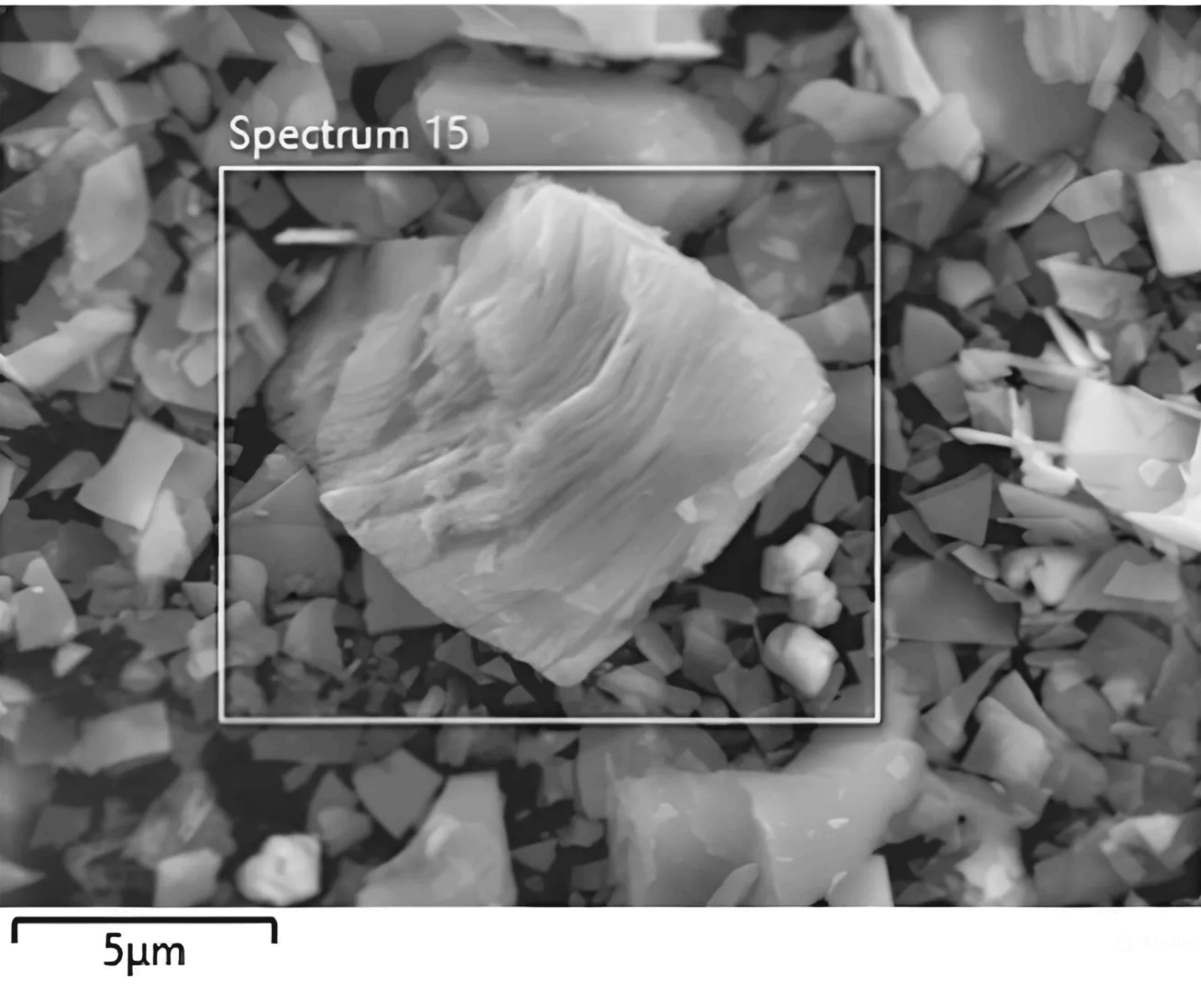
SEM Analysis of Ti3C2-BiOCl SEM: Scanning Electron Microscopy, Ti_3_C_2_-BiOCl: Titanium Carbide(MXene)-Bismuth Oxychloride.

The SEM, utilizing electrons rather than light, generates highly magnified images. Energy Dispersive X-ray Spectroscopy (EDS) complements SEM by providing localized chemical analysis via the X-ray spectrum emitted when a solid sample is bombarded with electrons. Combining SEM with EDS enables not only electron imaging but also element mapping and point analysis. Analysis of SEM images of the prepared samples reveals accordion-like multilayer structures in Ti_3_C_2_-BiOCl, characterized by thin sheet-like morphology and numerous pores. EDS, leveraging the X-ray spectrum emitted during electron bombardment, facilitates localized chemical analysis, enhancing our understanding of the material's composition (Figure 2).

Energy dispersive X-ray spectroscopy (EDS) analysis

**Figure 3:**
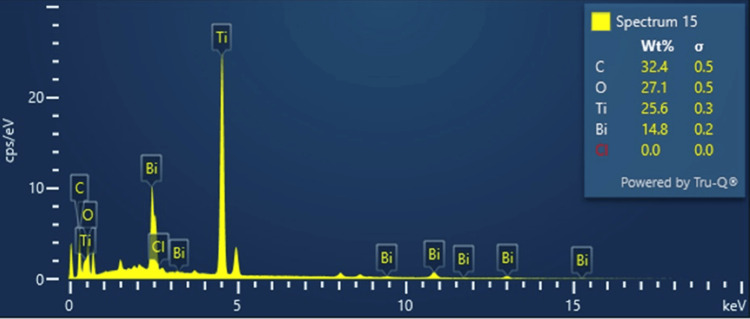
EDS Analysis of Ti3C2-BiOCl EDS: Energy Dispersive X-ray Spectroscopy, Ti_3_C_2_-BiOCl: Titanium Carbide(MXene)-Bismuth Oxychloride Carbon (C), Oxygen (O), Titanium (Ti), Bismuth (Bi), and Chloride (Cl).

EDS or EDAX, is an X-ray method used to detect and analyse the elements in the Ti_3_C_2_-BiOCl sample. Ti_3_C_2_ is primarily made up of titanium and carbon. EDS spectrum exhibits distinct X-ray peaks that correspond to the elements present. The intensities of these peaks may be utilized to determine the relative abundance of each element. The EDS mapping results (Figure 3) show a uniform dispersion of four elements Carbon (C), Oxygen (O), Titanium (Ti), Bismuth (Bi), and Chloride (Cl) in the Ti_3_C_2_-BiOCl compound. The weight percentages of C, O, Ti, Bi, and Cl were 32.45%, 27.12%, 25.64%, 14.79%, and 0.0%, respectively. The total weight of Ti_3_C_2_-BiOCl, being 100%, demonstrates its purity and the absence of impurities in the synthesized compound. EDS analysis confirms that no additional materials are present, indicating that the Ti_3_C_2_-BiOCl is impurity-free (Table 1). 

**Table 1:** EDS Spectrum Analysis of Ti3C2-BiOCl EDS: Energy Dispersive X-ray Spectroscopy, Ti_3_C_2_-BiOCl: Titanium Carbide(MXene)-Bismuth Oxychloride, Carbon (C), Oxygen (O), Titanium (Ti), Bismuth (Bi), and Chloride (Cl).

Element	Line Type	Apparent Concentration	k Ratio	Wt%	Wt% Sigma	Standard Label	Factory Standard
C	K Series	2.43	0.02428	32.45	0.48	C Vit	Yes
O	K Series	2.32	0.00779	27.12	0.47	SiO2	Yes
Cl	K Series	0.00	0.00000	0.00	0.04	NaCl	Yes
Ti	K Series	8.15	0.08154	25.64	0.25	Ti	Yes
Bi	M Series	4.63	0.04633	14.79	0.24	Bi	Yes
Total				100.00			

Bovine serum albumin (BSA) analysis

**Figure 4:**
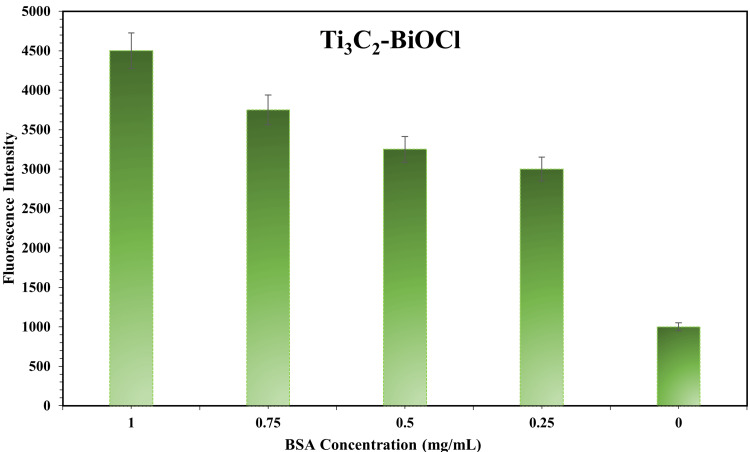
BSA Analysis of Ti3C2-BiOCl BSA: Bovine Serum Albumin, Ti_3_C_2_-BiOCl: Titanium Carbide(MXene)-Bismuth Oxychloride.

BSA is serum albumin sourced from bovine or cows. Its concentration denotes the amount of BSA dissolved within a specific volume of solution, typically expressed in grams per litre (g/L) or milligrams per millilitre (mg/mL). In an experiment, Ti_3_C_2_ -BiOCl was subjected to incubation for 12 h at -4ºC, followed by coating with BSA. The fluorescence intensity was measured at various BSA concentrations: 1.00 BSA concentration yielded a fluorescence intensity of 4500, 0.75 BSA concentration resulted in 3750, 0.50 BSA concentration exhibited 3250, 0.25 BSA concentration showed 3000, and 0.00 BSA concentration displayed a fluorescence intensity of 1000. Through this BSA concentration test, we have demonstrated that Ti_3_C_2_-BiOCl exhibits excellent sensitivity (Figure 4).

## Discussion

These nanomaterials exhibit significant promise across various applications, notably in the areas of antimicrobial and antioxidant properties [15]. In our endeavour to create these innovative nanoparticles, we embarked on a journey to synthesize two-dimensional Ti_3_C_2_-BiOCl particles using a straightforward yet effective method. Our synthesis approach prioritized precision and reproducibility, culminating in the production of nanoparticles that aligned with our expectations [16]. To validate the structural integrity and nanoscale morphology of these particles, we employed a comprehensive array of characterization techniques, including XRD, SEM, Transmission Electron Microscope (TEM), and EDS [17]. These analytical methods enabled us to confirm the successful formation of Ti_3_C_2_-BiOCl nanoparticles, verifying their desired morphology and elemental composition. Our findings not only underscore the feasibility of our synthesis methodology but also highlight its potential for scalability, paving the way for potential mass production and positioning it as a versatile tool in the realm of nanomaterial synthesis. One of the most notable findings from our investigation was the revelation of the remarkable antimicrobial properties displayed by Ti_3_C_2_-BiOCl nanoparticles [18]. These nanoparticles showcased an extraordinary capability to impede the growth of diverse microorganisms, spanning bacteria and fungi alike. Their mechanism of action involves the disruption of microbial membranes, a pivotal process in combating infections. This discovery holds profound significance in light of the escalating challenge of antibiotic resistance, as Ti_3_C_2_-BiOCl nanoparticles present a potential alternative for effectively combating microbial infections. The capacity to hinder the proliferation of harmful pathogens represents a promising pathway for the advancement of next-generation antimicrobial agents [19].

In addition to their effectiveness as antimicrobial agents, Ti_3_C_2_-BiOCl nanoparticles have demonstrated an unexpected yet valuable trait: remarkable antioxidant properties. These properties were evidenced by their ability to effectively scavenge free radicals, molecules known for their detrimental impact on cellular structures, thereby shielding cells from oxidative damage [20]. The implications of this discovery extend to the field of medicine, where the development of antioxidant-based therapies holds significant importance. Conditions characterized by oxidative stress, such as neurodegenerative disorders and cardiovascular diseases, could potentially benefit from the integration of Ti_3_C_2_-BiOCl nanoparticles into treatment strategies. The multifaceted functionality of these nanoparticles underscores their versatility and their potential to make substantial contributions to healthcare and overall well-being [21]. The antibacterial activity of Ti_3_C_2_Tx against Gram-positive *Bacillus subtilis* and Gram-negative* Escherichia coli *was evaluated by exposing bacteria to increasing concentrations of Ti_3_C_2_Tx colloidal solutions. The growth curve and cell viability were measured by spectrophotometrically assessing the optical density (OD) at 600 nm over various time intervals, spanning from the lag phase, where individual bacteria acclimate to their environment, to the stationary phase, where growth and death rates reach equilibrium. Subsequently, following a 4 h treatment with different concentrations of Ti_3_C_2_Tx, bacteria at a concentration of 107 colony-forming units (CFU)/mL were recultivated on agar plates and quantified using the bacteria counting method [22]. 

A secondary assay was conducted to validate the antibacterial efficacy of Ti_3_C_2_Tx nanosheets, employing bacterial regrowth curves. Growth curves of *B. subtilis* and *E. coli* cells cultured with varying doses of Ti_3_C_2_Tx were analysed. It was observed that bactericidal activity increased with higher Ti_3_C_2_Tx concentrations, and the suppression of both bacterial strains' growth was dose-dependent, as evidenced by the number of colonies formed on LB plates. Subsequently, growth kinetics constants for each bacterial strain were evaluated. Specifically, the specific growth constant (μe) for E. coli decreased from 0.277 h^-1^ to 0.068 h^-1^ as the concentration of Ti_3_C_2_Tx increased from 0 to 200 μg/mL. The bacterial doubling times (Td) for *E. coli *and *B. subtilis *increased from 2.5 to 10.11 h and from 2.0 to 5.16 h, respectively, with increasing Ti_3_C_2_Tx concentration from 0 to 200 μg/mL, indicative of significant bactericidal activity [23]. While significant progress has been made in understanding the antimicrobial and antioxidant properties of Ti_3_C_2_-BiOCl nanoparticles, the precise mechanisms governing these actions remain a subject of ongoing investigation. We hypothesize that the unique surface chemistry of the nanoparticles plays a pivotal role in their interactions with microbial cell walls and their ability to combat oxidative stress-inducing species. However, to fully exploit the potential of these nanoparticles, additional studies are required to elucidate the intricate mechanisms involved. Furthermore, exploring potential synergistic effects in combination with other materials or drugs may reveal new dimensions of their utility [24].

As we explore the myriad possibilities offered by Ti_3_C_2_-BiOCl nanoparticles, several exciting avenues for future research emerge. First and foremost, comprehensive studies into the biocompatibility of these nanoparticles within biological systems, including thorough in vivo investigations, are essential. Ensuring their safety is paramount for potential medical applications, necessitating further exploration in this area [25]. Moreover, investigating the potential for functionalizing Ti_3_C_2_-BiOCl nanoparticles holds promise in enhancing their selectivity and efficacy. This could be particularly valuable in the realm of targeted drug delivery, where precision is critical. Finally, the translation of scientific discoveries into practical applications often relies on the development of scalable production methods. Assessing the economic feasibility and practicality of commercializing Ti_3_C_2_-BiOCl nanoparticles represents a crucial step towards realizing their full potential in various fields [26].

Limitation

Ti_3_C_2_-BiOCl nanoparticles shows the low adsorption capacity to hydrophobic contaminants, high aggregation tendency and difficulty of separation and recovery. Optimization of the synthesis method to enhance yield, repeatability, and scalability of the fabrication process. Investigation of surface modification strategies to improve the stability, biocompatibility, and targeting abilities of Ti_3_C_2_-BiOCl nanomaterial linked oxides. Exploration of potential synergies by combining Ti_3_C_2_-BiOCl nanomaterials with other therapeutic methods, such as radiation therapy, immunotherapy, or chemotherapy. These efforts could contribute to the development of more effective and versatile cancer treatment strategies in the future.

## Conclusions

The investigation of recently developed two-dimensional Ti_3_C_2_-BiOCl nanoparticles shows they have excellent potential for antibacterial and antioxidant qualities. Ti_3_C_2_ MXene and BiOCl nanoparticles work synergistically to improve performance, making them extremely efficient against various microbial infections and conditions associated with oxidative stress. This creative method expands the range of applications for Ti3C2 MXene and BiOCl and creates new opportunities for developing cutting-edge materials for environmental remediation and healthcare.
